# Mutation analysis of the *COL1A1* and *COL1A2* genes in Vietnamese patients with osteogenesis imperfecta

**DOI:** 10.1186/s40246-016-0083-1

**Published:** 2016-08-12

**Authors:** Binh Ho Duy, Lidiia Zhytnik, Katre Maasalu, Ivo Kändla, Ele Prans, Ene Reimann, Aare Märtson, Sulev Kõks

**Affiliations:** 1 Hue University of Medicine and Pharmacy, Hue University, 06 Ngo Quyen, Hue city, 530000 Vietnam; 2 Department of Traumatology and Orthopaedics, University of Tartu, Puusepa 8, 51014 Tartu, Estonia; 3 Clinic of Traumatology and Orthopaedics, Tartu University Hospital, Puusepa 8, 51014 Tartu, Estonia; 4 Centre of Translational Medicine, University of Tartu, Ravila 14a, Tartu, 50411 Estonia; 5 Department of Pathophysiology, University of Tartu, Ravila 19, Tartu, 50411 Estonia

**Keywords:** Osteogenesis imperfecta, Collagen type I, Bone fragility, Sanger sequencing

## Abstract

**Background:**

The genetics of osteogenesis imperfecta (OI) have not been studied in a Vietnamese population before. We performed mutational analysis of the *COL1A1* and *COL1A2* genes in 91 unrelated OI patients of Vietnamese origin. We then systematically characterized the mutation profiles of these two genes which are most commonly related to OI.

**Methods:**

Genomic DNA was extracted from EDTA-preserved blood according to standard high-salt extraction methods. Sequence analysis and pathogenic variant identification was performed with Mutation Surveyor DNA variant analysis software. Prediction of the pathogenicity of mutations was conducted using Alamut Visual software. The presence of variants was checked against Dalgleish’s osteogenesis imperfecta mutation database.

**Results:**

The sample consisted of 91 unrelated osteogenesis imperfecta patients. We identified 54 patients with *COL1A1/2* pathogenic variants; 33 with *COL1A1* and 21 with *COL1A2*. Two patients had multiple pathogenic variants. Seventeen novel *COL1A1* and 10 novel *COL1A2* variants were identified. The majority of identified *COL1A1/2* pathogenic variants occurred in a glycine substitution (36/56, 64.3 %), usually serine (23/36, 63.9 %). We found two pathogenic variants of the *COL1A1* gene c.2461G > A (p.Gly821Ser) in four unrelated patients and one, c.2005G > A (p.Ala669Thr), in two unrelated patients.

**Conclusion:**

Our data showed a lower number of collagen OI pathogenic variants in Vietnamese patients compared to reported rates for Asian populations. The OI mutational profile of the Vietnamese population is unique and related to the presence of a high number of recessive mutations in non-collagenous OI genes. Further analysis of OI patients negative for collagen mutations, is required.

## Background

Osteogenesis imperfecta (OI) is associated with high genetic heterogeneity. To date, mutations in 16 different genes have been found to cause OI phenotypes of varying severity [1]. About 90 % of the mutations are related to alterations in the *COL1A1* and *COL1A2* genes, located at chromosome 17q21.33 and 7q21.3, respectively [2, 3]. These genes code for the α1/α2 chains of type 1 collagen [1, 4]. It was hypothesized that due to the presence of two α1 and one α2 chains in the procollagen triple helix, the *COL1A1* is more susceptible to mutation, as more α1 chains are implemented in the collagen fibrils. *COL1A1* gene mutations are more pathogenic and cause OI more often than *COL1A2* gene mutations. One third of glycine (Gly) substitutions in the *COL1A1* gene are lethal, whereas only 1/5 of Gly pathogenic variants in the *COL1A2* gene are fatal [5]. The collagen primary structure differs with an obligatory presence of Gly residues, the smallest amino acid, in every third position of an α chain, composing (Gly-X-Y)_n_ repetitions, where X and Y are random amino acids [6]. The substitution of Gly positioned in the center of the triple helix by a different amino acid would prevent interchain hydrogen bond formation between the NH-group of Gly and the CO-group in the X-position of a neighboring chain. Moreover, substitution of Gly residues with branched nonpolar or charged amino acids changes the helix to bulky and unstructured [5]. In this way, helix strength and stability decrease, which are crucially important for protein function [6–8].

Type 1 collagen is one of the most abundant proteins in the human body. It is a structural component of the bone, skin, tendons, cornea, and blood vessel walls and other connective tissues [4]. OI is generally caused by qualitative or quantitative collagen type I defects [9]. More than 2500 OI mutations have been found in type I collagen genes, which can cause a wide range of OI phenotypes that range in severity from mild to severe [10, 11] (http://www.le.ac.uk/ge/collagen/). Previous studies have shown that *COL1A1/2* mutations account for up to 85–90 % of all OI causative mutations, whereas only 10–15 % of OI mutations occur in non-collagenous genes [2, 11, 12]. While in more recent studies, many new genetic causes have been described, the mutations in the *COL1A1/2* genes remain a common origin of OI [1, 10]. However, there is a lack of systematic information regarding the mutational characteristics of OI patients. In addition, the genetics of Vietnamese OI patients has not been studied before. Our main aim with the current study was to perform mutational analysis of the *COL1A1* and *COL1A2* genes among unrelated OI patients of Vietnamese origin. We applied a systematic approach to characterizing the mutation profiles of these two genes.

## Materials and methods

The study was conducted in accordance with the Helsinki Declaration and received approval from the ethical review board of Hue University Hospital (approval no. 75/CN-BVYD) and the Ethical Review Committee on Human Research of the University of Tartu (permit no. 221/M-34). Patients were selected from the Vietnamese database of osteogenesis imperfecta patients. The database includes information on 146 OI patients from 120 OI families and also about their healthy family members. A total of 91 unrelated OI patients were included in the study. Informed written consent from the patients or their legal representatives was obtained prior to inclusion to the study. Investigators then contacted patients in order to conduct an interview, perform a clinical examination, and collect blood samples, including blood samples from parents, siblings, and close relatives. Genomic DNA was extracted from EDTA-preserved blood according to standard high-salt extraction methods, stored at −80 °C, and analyzed at the University of Tartu, Estonia.

DNA samples were amplified using a polymerase chain reaction (PCR) with 25 specially designed primer pairs covering the 5′ UTR and 3′ UTR regions and 51 exons of the *COL1A1* gene; 36 primer pairs covering the 5′ UTR and 3′ UTR regions and 52 exons of the *COL1A2* gene. The PCR reaction was performed in a total volume of 20 μl, which included 4 μl of 5× HOT FIREPol® Blend Master Mix Ready to Load with 7.5 mM MgCl_2_ (Solis BioDyne, Estonia), 1 μl each of forward and reverse primer (5 pmol), and 1 μl of gDNA (50 ng). PCR reaction was performed with a Thermal Cycler (Applied Biosystems, USA) PCR machine. The PCR *touchdown* program was used as follows for the reaction of amplification:

- 1 = 95.0°; 15:00 min
- 2 = 95.0°; 0:25 min
- 3 = 64.0°; 0:30 min
- 4 = 72.0°; 0:40 min
- 5 = go to 2.4 times
- 6 = 95.0°; 0:25 min
- 7 = 62.0°; 0:30 min
- 8 = 72.0°; 0:40 min
- 9 = go to 6.30 times
- 10 = 72.0°; 5:00 min
- 11 = 6.0°; forever

Amplified PCR products were electrophoresed through a 1.5 % agarose gel, to control the quality of fragments. The PCR products then purified with exonuclease I and shrimp alkaline phosphatase (Thermo Fisher Scientific, USA). Sanger sequencing reactions were performed on the purified PCR fragments using a BigDye® Terminator v3.1 Cycle Sequencing Kit (Applied Biosystems, USA). Reactions were processed on the ABI3730xl instrument.

Sequence reads were analyzed using Applied Biosystems’ Sequence Scanner v1.0 and aligned to the human reference genome Local Reference Genomic sequence LGR_1 and GR_2. Raw sequencing data are available from authors upon request. Sequence analysis and pathogenic variant identification were performed with Mutation Surveyor DNA variant analysis software (Softgenetics, USA). Prediction of mutation’s pathogenicity was performed using Alamut Visual software (Interactive Biosoftware, France). Variants were checked against the osteogenesis imperfecta mutation database (http://www.le.ac.uk/ge/collagen/). The pathogenicity of the pathogenic variants was predicted with SIFT score [13].

## Results

We studied 42 female and 49 male OI patients. To characterize the OI patients’ clinical features, all participants underwent clinical and physical examinations, and their medical records were reviewed. Cases were described according to the Sillence classification (types I–IV) [14].

**Fig. 1:**
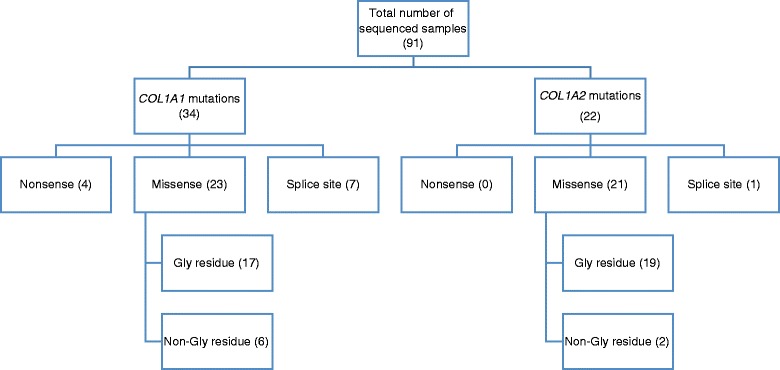
Type I collagen OI mutations present among the studied Vietnamese OI patients

Fifty-four patients were found to have *COL1A1/2* mutations, 33 with *COL1A1* and 21 with *COL1A2*; this equated to 36.3 and 23.1 % of patients, respectively, totaling 59.4 % of the studied OI cases exhibiting collagen type I mutations. Thirty-four pathogenic variants in the *COL1A1* gene (missense = 23, nonsense = 4, splice site = 7) and 22 pathogenic variants in the *COL1A2* gene (missense = 21, splice site = 1) were identified (patients VN01 and VN47 were carriers of double pathogenic variants in both the *COL1A1/2* genes) (Fig. 1; Tables 1 and 2). According to Dalgliesh database, 17 *COL1A1* and 10 *COL1A2* variants have not been reported before (Tables 1 and 2). De novo mutations were observed in 50 % (17/34) of *COL1A1* variants and 45.5 % (10/22) of *COL1A2* variants. All mutations were highly pathogenic, with a SIFT score of 0.0 and rarely 0.1, and located in regions of high conservation.

**Table 1:** *COL1A1* mutations in unrelated Vietnamese OI patients

No	Patient ID	COL1A1 mutation	Exon	Mutation type	Protein alteration	Sillence OI type
1	VN01	c.2461G > GA	Exon 37	Missense	p.Gly821Ser	I
		c.2005G > GA*	Exon 30	Missense	p.Ala669Thr,	
2	VN02	c.1200 + 1G > GT*	Intron 18	Splice site	–	I
3	VN05	c.1072delC, het*	Exon 17	Frameshift	p.Glu358Lysfs*26	III
4	VN13	c.4391 T > C	Exon 52	Missense	p.Leu1464Pro	I
5	VN18	c.103 + 2 T > TC*	Intron 1	Splice site	–	IV
6	VN21	c.4352dupA, het.*	Exon 52	Nonsense Frameshift	p.Asp1451Glufs*100	IV
7	VN26	c.3226G > GA	Exon 45	Missense	p.Gly1076Ser	IV
8	VN34	c.2461G > GA	Exon 37	Missense	p.Gly821Ser,	IV
9	VN38	c.959G > GA*	Exon 15	Missense	p.Gly320Asp	IV
10	VN39	c.630delG, het*	Exon 8	Frameshift	p.Glu210Aspfs*3	III
11	VN40	c.2461G > GA	Exon 37	Missense	p.Gly821Ser	IV
12	VN41	c.1102G > GA	Exon 17	Missense	p.Gly368Ser	IV
13	VN49	c.2461G > GA	Exon 37	Missense	p.Gly821Ser	IV
14	VN50	c.932G > GT*	Exon 14	Missense	p.Gly311Val	III
15	VN51	c.949G > GA*	Exon 14	Missense	p.Gly317Ser	IV
16	VN52	c.2523delT, het.	Exon 37	Frameshift Nonsense	p.Gly842Alafs*266	I
17	VN58	c.2236-2A > AG*	Intron 32	Splice site	-	I
18	VN66	c.2596G > AG*	Exon 38	Missense	p.Gly866Ser	III
19	VN68	c.2299G > GA	Exon 33/34	Missense	p.Gly767Ser	I
20	VN70	c.2281G > GA*	Exon 33/34	Missense	p.Gly761Ser	IV
21	VN71	c.1002 + 2 T > C	Intron 15	Splice site	–	IV
22	VN72	c.1165G > GT	Exon 18	Missense	p.Gly389Cys	I
23	VN76	c.1165G > GA	Exon 18	Missense	p.Gly389Ser	III
24	VN78	c.3766G > GA	Exon 49	Missense	p.Ala1256Thr	I
25	VN86	c.977G > AG	Exon 15	Missense	p.Gly326Asp	I
26	VN88	c.2005G > GA*	Exon 30	Missense	p.Ala669Thr	IV
27	VN89	c.2005G > GA*	Exon 30	Missense	p.Ala669Thr	IV
28	VN91	c.1299 + 1G > C	Intron 19	Splice site	–	IV
29	VN92	c.2299G > GA	Exon 33/34	Missense	p.Gly767Ser	III
30	VN95	c.590G > GA	Exon 8	Missense	p.Gly197Asp	I
31	VN99	c.103 + 2 T > TC*	Intron 1	Splice site	–	I
32	VN104	c.3369 + 1G > GC*	Intron 46	Splice site	–	I
33	VN106	c.1350G > GC*	Exon 20	Missense	p.Glu450Asp	III

Mutations unreported in the Dalgliesh’s OI database are marked with an *asterisk* (*). In the case of heterozygous mutation, both the wild type and mutated alleles are indicated after an *arrow* (>)

**Table 2:** *COL1A2* mutations in unrelated Vietnamese OI patients

	Patient ID	COL1A2 mutation	Exon	Mutation type	Protein alteration	Sillence OI type
1	VN09	c.3305G > GT	Exon 49	Missense	p.Gly1102 > Val	I
2	VN23	c.2261G > GT*	Exon 37	Missense	p.Gly754Val	III
3	VN25	c.1072G > GT	Exon 37	Missense	p.Gly358Ser	I
4	VN29	c.1630G > GA*	Exon 28	Missense	p.Gly544Ser	IV
5	VN45	c.1090G > GA	Exon 21	Missense	p.Gly364Ser	III
6	VN47	c.3034G > GA	Exon 46	Missense	p.Gly1012Ser	IV
		c.2569C > CA	Exon 41	Missense	p.Pro857Thr	
7	VN48	c.1451G > GA	Exon 25	Missense	p.Gly484Glu	IV
8	VN56	c.1729G > GA*	Exon 30	Missense	p.Gly577Ser	III
9	VN60	c.1009G > GA	Exon 19	Missense	p.Gly337Ser	IV
10	VN62	c.1378G > GA	Exon 24	Missense	p.Gly460Ser	IV
11	VN64	c.1964G > GT*	Exon 32	Missense	p.Gly655Val	IV
12	VN65	c.1981G > GC*	Exon 33	Missense	p.Gly661Ser	III
13	VN69	c.874G > GA	Exon 17	Missense	p.Gly292Ser	III
14	VN81	c.982G > GA	Exon 19	Missense	p.Gly328Ser	III
15	VN82	c.2503G > GA	Exon 40	Missense	p.Gly835Ser	III
16	VN83	c.792 + 1G > GA	Exon 16	Splice site	-	III
17	VN84	c.2791G > GA*	Exon 43	Missense	p.Gly931Arg	IV
18	VN85	c.838G > GT*	Exon 17	Missense	p.Gly280Cys	IV
19	VN87	c.2791G > GA*	Exon 43	Missense	p.Gly931Arg	IV
20	VN96	c.892G > GT*	Exon 18	Missense	p.Gly298Cys	III
21	VN97	c.2538G > GT*	Exon 40	Missense	p.Lys846Asp	I

Mutations unreported in the Dalgliesh’s OI database are marked with an *asterisk* (*). In the case of heterozygous mutation, both the wild type and mutated alleles are indicated after an *arrow* (>)

## Discussion

In our study, we performed mutational analysis of 91 Vietnamese patients clinically diagnosed with OI (types I–IV). Thirty-three patients had 34 pathogenic variants of the *COL1A1* gene, and 21 patients had 22 pathogenic variants of the *COL1A2* gene, equating to a total of 54/91 (59.4 %) patients with *COL1A1/2* pathogenic variants. Previous studies have indicated that nearly 90 % of all OI mutations appear in the *COL1A1* and *COL1A2* genes [12, 15]. However, reported collagen type I mutational rates vary between different populations from 58 to 96 % [16–18].

We identified the substitution of Gly residuals in 17 out of 23 missense *COL1A1* mutations and 19 of 21 missense *COL1A2* pathogenic variants. Gly substitutions composed 36/56 (64.3 %) of *COL1A1/2* pathogenic variants. It has been hypothesized that the majority of the clinically severe forms of OI are caused by Gly missense mutations [17, 18]. However, there may exist a complex relationship between OI pathogenic variant and OI severity, whereby genetic, epigenetic, and environmental factors altogether affect the phenotype [19, 20].

Our research showed that out of 36 glycine substitutions, serine was the most prevalent (23/36; 63.9 %), followed by valine (4/36; 11.1 %), and cysteine and aspartic acid (3/36 cases each). Previous studies have suggested that glycine substitutions by cysteine often cause a greater severity of OI phenotype, and glycine substitutions by arginine were often fatal [21]. However, there are alternative reports that also suggest serine is the most common substitutional residue of Gly (72 % among Chinese OI patients) [18]. Aspartic acid substituted Gly in 40 % of Taiwanese OI patients [22]. The cause of variation in amino acid substitutions among populations of different geographical regions is still unclear.

In our research, intronic variants were represented by seven splice site mutations; other research has reported intronic variants among 7/56 of Chinese OI patients [18]. These mutations may cause exon skipping, intronic inclusion, and activation of cryptic sites [23]. In addition, analyses identified two nonsense mutations located in exons 52 and 37. Nonsense and splice site mutations are associated with haploinsufficiency, and as a result, quantitative collagen type I defects and a mild–moderate OI phenotype (type I/IV).

Patients VN01, VN34, VN40, and VN49 had the same heterozygous mutation: c.2461G > A (p.Gly821Ser) in exon 37 of the *COL1A1* gene. With respect to clinical severity, these patients showed nearly the same manifestations (clinical types I and IV). However, previous studies have described OI patients with different clinical features, despite their being carriers of the c.2461G > A mutation. Current data highlights the complexity of OI genotype–phenotype correlations. It is not yet possible to predict disorder severity based only on mutational analysis data.

Families VN88 and VN89 shared the same heterozygous *COL1A1* c.2005G > A (p.Ala669Thr) pathogenic variant in exon 30. Two patients had the same pathogenic variant and level of OI severity (type IV). Similar cases of variant reoccurance have been described before by Zhang et al. and Lee et al. in both *COL1A1/2* genes [17, 18]. However, OI pathogenic variants are usually unique and rarely repeated among different families [17].

Genetic analysis revealed the presence of two heterozygous *COL1A1* mutations: exon 37 c.2461G > A (p.Gly821Ser) and exon 30 c.2005G > A (p.Ala669Thr) in patient VN01. Both pathogenic variants were shared by other unrelated patients among our study cohort. Patient VN47 had two heterozygous *COL1A2* pathogenic variants: exon 46 c.3034G > GA (p.Gly1012Ser) and exon 41 c.2569C > CA (p.Pro857Thr). Takagi et al. reported one case of severe OI (types II–III) due to a double substitution of glycine residues in the *COL1A2* gene (p.Gly208Glu and p.Gly235Asp), located on the same allele [24]. Our patients had only one substituted Gly residue in the *COL1A1* gene and a mild phenotype (VN01) and moderate phenotype (VN47) based on the clinic examination.

Of the 56 mutations found during our research, 17 *COL1A1* and 10 *COL1A2* variants (27/56 pathogenic variants; 48.2 %) were not present in Dalgleish’s OI mutation database (Tables 1 and 2) [https://oi.gene.le.ac.uk/home.php?select_db=COL1A1; https://oi.gene.le.ac.uk/home.php?select_db=COL1A2]. The percentage of new variants among our patients was higher than in previous studies [16, 22, 25, 26]. The novelty of the pathogenic variants highlights the originality of the genetic epidemiology of the Vietnamese OI population. Half of Vietnamese OI patients are carriers of rare recessive non-collagenous OI pathogenic variants, which will be further identified with the whole exome sequencing analysis and reported in a future paper.

**Fig. 2:**
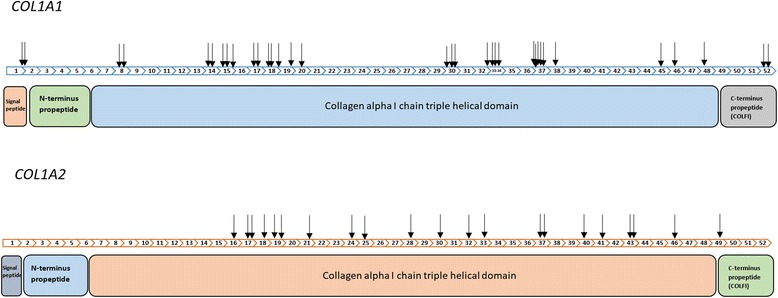
Diagram of the *COL1A1* and *COL1A2* exons, with identified mutations and corresponding to protein product domains

According to our data, more OI causative pathogenic variants occurred in the *COL1A1* gene than the *COL1A2* gene. Mutation hotspots were observed in intron 1; exons 8, 14–15, 17–20, 30, 33, 34, 37, and 52 of the *COL1A1* gene; and exons 17–49 of the *COL1A2* gene (Fig. 2). Products of the *COL1A1/2* gene consisted of signal peptide, N-terminal propeptide, collagen alpha I/II chain triple helical domain, and C-terminal propeptide (COLFI). COLFI controls procollagen intracellular assembly and the extracellular assembly of collagen fibrils. Mutation hotspots were situated in the regions that tolerate amino acid substitutions, and pathogenic variant resulted in an altered protein, but the organisms were still able to survive. Gaps in the mutation map connected to regions with crucial functions can however lead to fatal alterations [5, 27].

Sequencing primers for the performed Sanger sequencing of the *COL1A1* and *COL1A2* genes in patients with clinical signs of osteogenesis imperfecta were designed far from intron-exon splice sites, which allowed the identifying of splice site, missense, frameshift, and nonsense mutations in the exons of the *COL1A1/2* genes. The gold standard of sequencing, the Sanger method, has an accuracy of approximately 99.9 % [28]. However, it has limitations in identifying whole genes and exon duplications and deletions. Therefore, the number of *COL1A1/2* pathogenic variants in the studied OI patients might have been underestimated.

We must also take into consideration that the percentage of collagen pathogenic variants among osteogenesis imperfecta patients may vary between studies due to their different sample sizes. However, we cannot exclude the possibility that the Vietnamese population has lower rates of collagenous OI pathogenic variants, and a unique OI mutational profile with higher levels of rare non-collagenous pathogenic variants, compared to other populations.

## Conclusion

In the current study, we conducted mutational analysis of the *COL1A1* and *COL1A2* genes among 91 Vietnamese patients with osteogenesis imperfecta. After sequencing of the *COL1A1* and *COL1A2* genes, we found 56 mutations in 54 patients (59.4 % of patients). Our data showed a lower number of collagen OI pathogenic variants in these Vietnamese patients compared to reported rates for other Asian OI populations. The OI mutational profile of the Vietnamese population is likely unique and is related to the presence of a high number of recessive mutations in non-collagenous OI genes. Further analysis of patients negative for collagen OI mutations is needed in order to reveal unidentified OI genotypes from the sample.

## Abbreviations

3′ UTR, 3′ untranslated region; 5′ UTR, 5′ untranslated region; COLFI, fibrillary collagen C-terminal domain; EDTA, ethylenediaminetetraacetic acid; gDNA, genomic DNA; OI, osteogenesis imperfecta; PCR, polymerase chain reaction
